# Supplementary material for: The molecular basis of immunosuppression by soluble CD52 is defined by interactions of N-linked and O-linked glycans with HMGB1 box B
Source: J Biol Chem. 2025 Feb 25;301(4):108350. doi: 10.1016/j.jbc.2025.108350 (PMC11982460; doi:10.1016/j.jbc.2025.108350)
Supplement: Table_S7 [file mmc7.pdf]

Table S7 GlyToucan Accessions ID's mentioned in main text.

Accession	Structure	Link
G52120NK	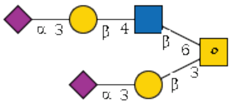	https://glytoucan.org/Structures/Glycans/G52120NK
G63110FE	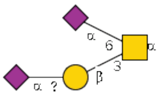	https://glytoucan.org/Structures/Glycans/G63110FE
G80552MJ	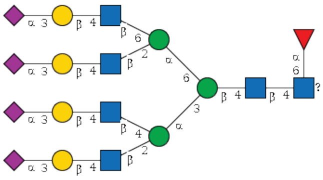	https://glytoucan.org/Structures/Glycans/G80552MJ
G42089IU	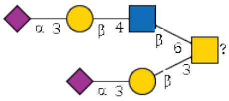	https://glytoucan.org/Structures/Glycans/G42089IU
G56655CC	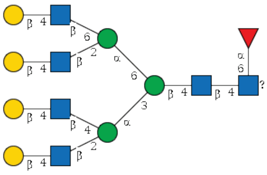	https://glytoucan.org/Structures/Glycans/G56655CC
G96017QA	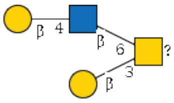	https://glytoucan.org/Structures/Glycans/G96017QA
