# Supplementary material for: The molecular basis of immunosuppression by soluble CD52 is defined by interactions of N-linked and O-linked glycans with HMGB1 box B
Source: J Biol Chem. 2025 Feb 25;301(4):108350. doi: 10.1016/j.jbc.2025.108350 (PMC11982460; doi:10.1016/j.jbc.2025.108350)
Supplement: Supp_Figure_with_legend_S1 [file mmc8.pdf]

Figure S1 Example MS/MS spectrum of tri-antennary, tetra-sialylated N-glycan containing a di-sialic acid.

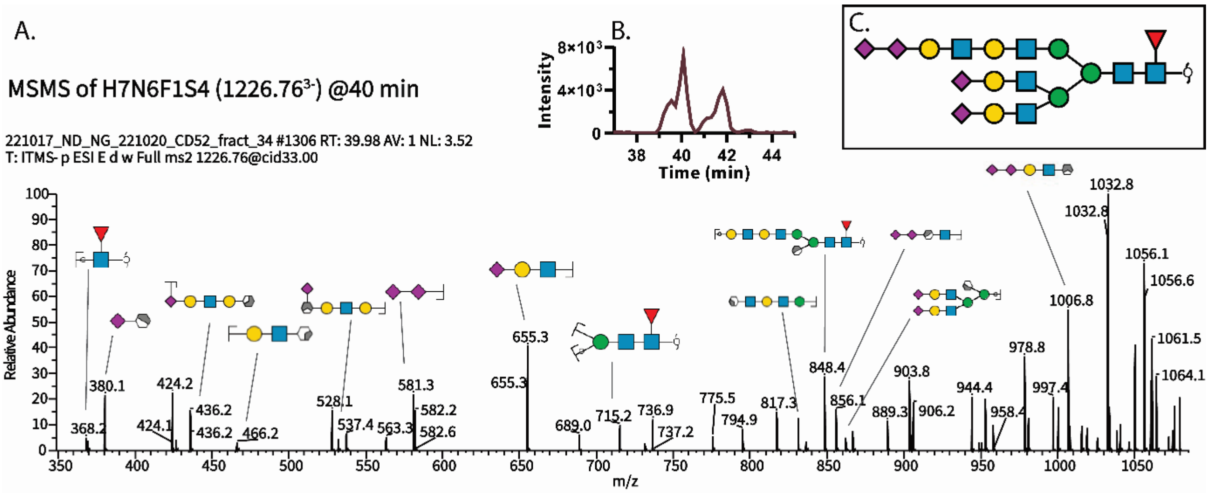

A) MS/MS analysis of the triply charged N-glycan isomer H7N6F1S4 (1226.76^{M-3H}) at 39 minutes. Several clear diagnostic glycan signatures provide clues as to the structure of this glycan, including m/z 368.2 (core fucosylation), 436.2 (sialyl-diLacNAc extension), and 581.3 (di-sialylation) among others. B) The elution profile of this glycan composition shows separation of two major isomers. It is possible that there are other isomers in these peaks which have not been sufficiently chromatographically resolved. C) Solved structure of the H7N6F1S4 glycan isomer eluting as the 40-minute peak.
