# Supplementary material for: The molecular basis of immunosuppression by soluble CD52 is defined by interactions of N-linked and O-linked glycans with HMGB1 box B
Source: J Biol Chem. 2025 Feb 25;301(4):108350. doi: 10.1016/j.jbc.2025.108350 (PMC11982460; doi:10.1016/j.jbc.2025.108350)
Supplement: Supp_Figure_with_legend_S2 [file mmc9.pdf]

Figure S2 **Example MS/MS spectrum of most abundant O-Glycan H2N2S2**

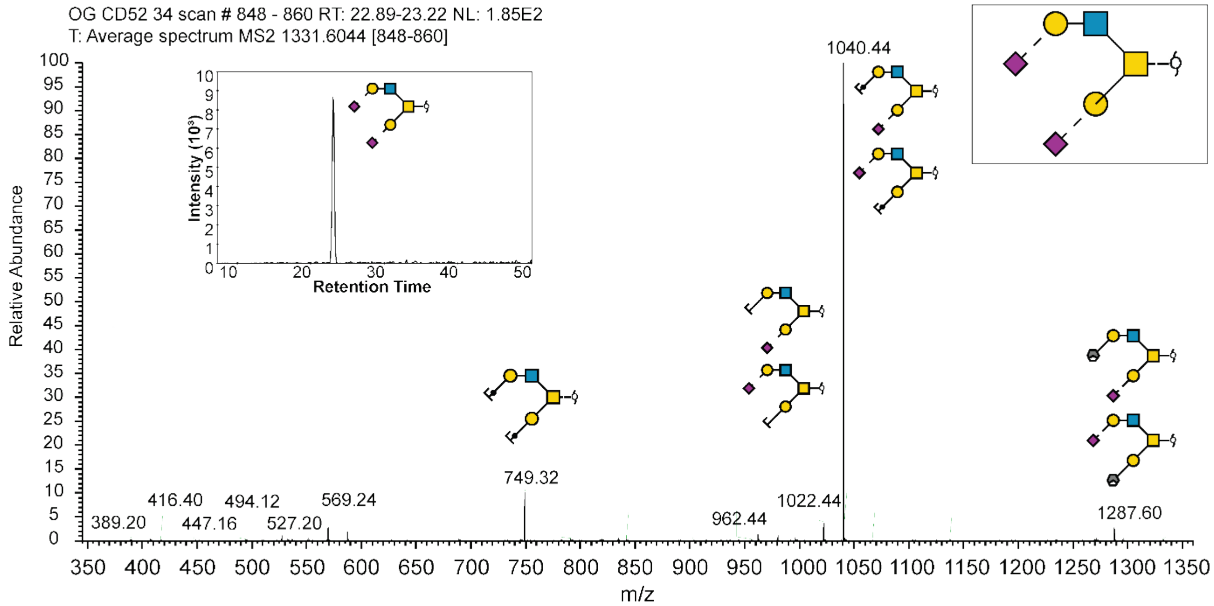

MSMS analysis of the singly charged H2N2S2 O-glycan (1331.6^{M-H}) at 22.9 minutes. Only a single isomer of this glycan was present in recombinant CD52 fractions. Sialylation linkage is inferred from prior knowledge of PGC retention time.
