# Supplementary material for: The molecular basis of immunosuppression by soluble CD52 is defined by interactions of N-linked and O-linked glycans with HMGB1 box B
Source: J Biol Chem. 2025 Feb 25;301(4):108350. doi: 10.1016/j.jbc.2025.108350 (PMC11982460; doi:10.1016/j.jbc.2025.108350)
Supplement: Supp_Figure_with_legend_S3 [file mmc10.pdf]

Figure S3 Analysis of radius of gyration of CD52 modelling both with (a) and without (b) terminal sialylation present on the 2 glycans modelled.

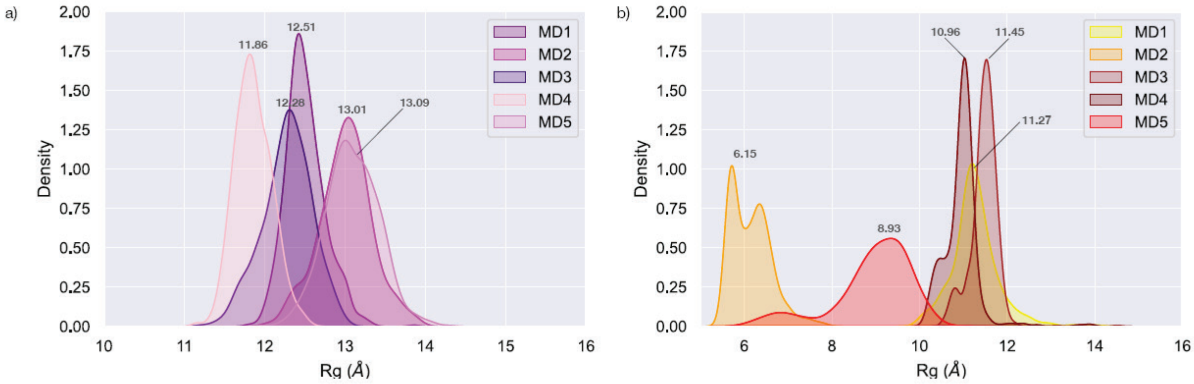

Analysis of radius of gyration of CD52 modelling both with (a) and without (b) terminal sialylation present on the 2 glycans modelled. Modelled glycans included an N-glycan on N3 (GlyTouCan ID G80552MJ or GlyTouCan ID G56655CC) and an O-glycan on T8 (GlyTouCan ID G96017QA or GlyTouCan ID G42089IU). With terminal siaylation present, glycosylated CD52 was modelled to be have a more uniform, extended position than CD52 that was lacking terminal sialylation.
