# Supplementary material for: The molecular basis of immunosuppression by soluble CD52 is defined by interactions of N-linked and O-linked glycans with HMGB1 box B
Source: J Biol Chem. 2025 Feb 25;301(4):108350. doi: 10.1016/j.jbc.2025.108350 (PMC11982460; doi:10.1016/j.jbc.2025.108350)
Supplement: Supp_Figure_with_legend_S5 [file mmc12.pdf]

Figure S5 Ramachandran plot of CD52 with all non-sialylated glycans modelled.

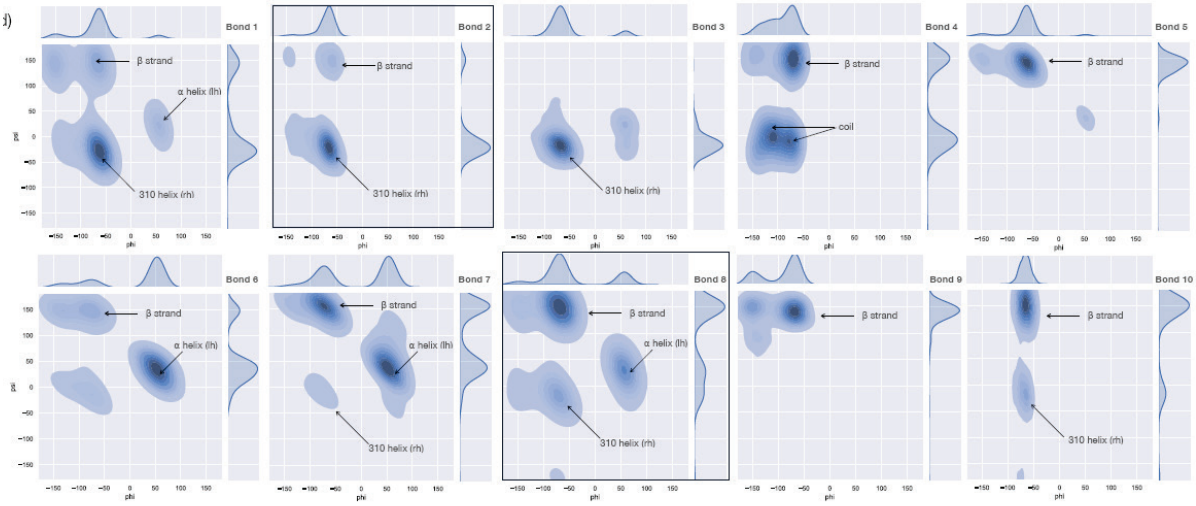

Ramachandran plot of CD52 with all non-sialylated glycans modelled (GlyTouCan ID G96017QA on T8 and GlyTouCan ID G56655CC on N3).
